# A Board of Guardians Heavily Fined

**Published:** 1899-11-18

**Authors:** 


### A BOARD OF GUARDIANS HEAVILY FINED.

At the North London Police-court the guardians of St. Leonard's, Shoreditch, were heavily fined recently for not conforming with the requirements of the London Building Act, 1894, in regard to certain buildings. The district surveyor said that in May last he found one of the old houses in Lower Clapton Road being used as a workhouse school. He required the guardians to make certain alterations, and put in a fireproof staircase. The matter, however, was contested; and, being brought before that Court, the nominal penalty of 1s. was imposed on the guardians. Since then, however, nothing had been done to meet the requirements of the Act, and as there had recently been a fire at the house, he now thought it his duty again to bring the matter before the Court. On behalf of the guardians, it was stated that plans had to be prepared and submitted to the Local Government Board; but the surveyor said that the staircase could have been put in in three weeks. On this the Magistrate, remarking that although five months had gone by nothing had been done, imposed a penalty of £2 a day for 25 days—£50, and costs. Probably these children now will get their staircase. As a commentary on this we may refer to a fire which took place the very day after this case was tried, when the workhouse at Westhampnett, in Sussex, was entirely destroyed, nothing being left but the walls and chimneys. The whole of the 115 inmates were successfully removed, but one old man expired soon afterwards from shock.

